# Neuropsychiatric Insights into Postpartum Psychosis: A Four-Case Series with CSF, EEG, and Neuroimaging Correlates

**DOI:** 10.1192/j.eurpsy.2026.10510

**Published:** 2026-07-31

**Authors:** S. Turkan, R. S. İlhan

**Affiliations:** Psychiatry, Ankara University, Ankara, Türkiye

## Abstract

**Introduction:**

Postpartum psychosis (PP) is a rare but severe psychiatric emergency, affecting 1–2 per 1000 births and associated with high risks of maternal suicide and infanticide (Munk-Olsen et al., NEJM, 2011). Traditionally considered within the affective spectrum, atypical features such as catatonia, cognitive impairment, and suicidality suggest broader neurobiological mechanisms. Emerging evidence links PP with neuroimmune dysregulation and metabolic vulnerability, yet systematic integration of cerebrospinal fluid (CSF), electroencephalogram (EEG), and neuroimaging remains scarce (Bergink et al., Am J Psychiatry, 2016).

**Objectives:**

We present four PP cases with multimodal biological evaluation to explore neurobiological contributors and treatment implications.

**Methods:**

Four women with acute-onset PP within two weeks postpartum were retrospectively analyzed. All underwent psychiatric evaluation, Bush–Francis Catatonia Rating Scale when relevant, EEG, brain MRI, and CSF analysis (cell count, protein, glucose, autoimmune panel including anti-NMDAR). Two also had FDG-PET scans. Treatments and six-month outcomes were recorded.

**Results:**

Case 1 (30y): Confabulation, visual hallucinations, memory deficits, catatonia (BFCRS=14). Catatonia improved with lorazepam. MRI/CSF/EEG unremarkable. Olanzapine 5 mg/day plus high-dose thiamine produced full remission of cognitive and perceptual symptoms.

Case 2 (28y): Suicide attempt, resistant hallucinations and persecutory delusions. EEG slowing; PET showed patchy parietal hypometabolism; CSF revealed increased immunoglobulins. Symptoms improved after IV methylprednisolone.

Case 3 (31y): Irritable mania with referential delusions. EEG slowing; PET revealed bilateral frontal and posterior cingulate hypometabolism; CSF non-specific. Remission achieved after eight ECT sessions plus quetiapine 300 mg/day.

Case 4 (39y): Bipolar history, persecutory delusions, aggression. CSF showed type 2 oligoclonal bands; EEG and PET were non-specific. Symptoms improved with eight ECT sessions plus lithium 600 mg/day and risperidone 4 mg/day.

EEG slowing was observed in three of four cases. All patients remained relapse-free at six months.

**Conclusions:**

This case series demonstrates the heterogeneity of PP, from catatonia and hallucinations to suicidality and mania. Multimodal evaluation revealed clinically meaningful abnormalities, including CSF immunoglobulin elevation, oligoclonal bands, and FDG-PET hypometabolism. Personalized interventions, including thiamine, corticosteroids, antipsychotics and ECT, proved decisive. To our knowledge, this is among the first PP series systematically integrating CSF, EEG, and PET findings with treatment outcomes. These results support neuroimmune and metabolic contributions and emphasize the need for prospective biomarker-driven studies to guide individualized management.

**Disclosure of Interest:**

None Declared

